# Investigation of the Chemical Composition, Microstructure, Density, Microhardness, and Elastic Modulus of the New β Ti-50Nb-xMo Alloys for Biomedical Applications

**DOI:** 10.3390/ma17010250

**Published:** 2024-01-03

**Authors:** José Roberto Severino Martins Junior, Pedro Akira Bazaglia Kuroda, Carlos Roberto Grandini

**Affiliations:** 1 Instituto Federal de São Paulo, Campus Caraguatatuba, Caraguatatuba 17607-220, SP, Brazil; jose.martins@ifsp.edu.br; 2 Laboratório de Anelasticidade e Biomateriais, UNESP—Universidade Estadual Paulista, Bauru 17033-360, SP, Brazil; carlos.r.grandini@unesp.br

**Keywords:** elastic modulus, hardness, materials characterization, Ti-50Nb-xMo alloys

## Abstract

β-type titanium alloys with a body-centered cubic structure are highly useful in orthopedics due to their low elastic modulus, lower than other commonly used alloys such as stainless steel and Co-Cr alloys. The formation of the β phase in titanium alloys is achieved through β-stabilizing elements such as Nb, Mo, and Ta. To produce new β alloys with a low modulus of elasticity, this work aimed to produce our alloy system for biomedical applications (Ti-50Nb-Mo). The alloys were produced by arc-melting and have the following compositions Ti-50Nb-xMo (x = 0, 3, 5, 7, and 12 wt% Mo). The alloys were characterized by density, X-ray diffraction, scanning electron microscopy, microhardness, and elastic modulus. It is worth highlighting that this new set of alloys of the Ti-50Nb-Mo system produced in this study is unprecedented; due to this, there needs to be a report in the literature on the production and structural characterization, hardness, and elastic modulus analyses. The microstructure of the alloys has an exclusively β phase (with bcc crystalline structure). The results show that adding molybdenum considerably increased the microhardness and decreased the elastic modulus, with values around 80 GPa, below the metallic materials used commercially for this type of application. From the produced alloys, Ti-50Nb-12Mo is highlighted due to its lower elastic modulus.

## 1. Introduction

The loss of some functions of the human body can occur through traumas of the most diverse reasons, originating from automobile accidents, firearms, work accidents, or even sports practices. Thus, using artificial parts or prostheses is often becoming necessary [1]. The biomaterials used in these repairs or restorations represent an adjustment in the characteristics and properties of the human body. The development of the biomaterials area took into account the appropriate combination of physical properties close to the replaced tissue with minimal toxic response to the foreign body [2]. Metallic materials that present this great combination are titanium and its alloys. Thus, these materials have been widely used in prostheses and special devices in the medical and dental areas since 1970 due to their properties as low values of elastic modulus (Young Modulus), high corrosion resistance, and biocompatibility characteristics [3,4,5]. However, the elastic modulus values of these materials are still about 2–4 times higher than those of human bone [6,7,8].

Among the most promising new materials for biomedical use are alloys of the Ti-Mo-Nb system because these alloys have exciting properties such as low elastic modulus, high resistance to corrosion, shape memory, superplasticity, and biocompatibility [9,10,11]. The Ti-Mo-Nb system was studied by Zhang et al. [12] using first-principle calculations and computational tools to obtain the shear and elastic modulus and Poisson’s ratio of these alloys. They also compared their results with experimental data from Ti-11.17Mo-10.82Nb, Ti-9.18Mo-26.68Nb, Ti-10.62Mo-20.55Nb, and Ti-10.11Mo-29.38Nb alloys, and the values obtained for the elastic modulus by the computational and experimental method are very close.

Borborema et al. [13] analyzed the influence of Nb content on the phases formed in the Ti-12Mo-Nb alloy (Nb = 0, 3, 8, 13, 17, and 20 wt%). The authors show that Nb acts as a stabilizing β element, suppressing the formation of the α” phase present in the Ti-12Mo alloy. On the other hand, the number of solutes used to form the Ti alloys promoted the formation of the metastable ω phase. According to the authors, the suppression of the α″ phase and the appearance of the ω phase, through the addition of Nb, causes a sharp increase in the elastic modulus; in addition, the decrease in the ω phase reduces the hardness of the Ti-12Mo-Nb system alloys.

Li-Juan Xu et al. [14] studied the Ti-15Mo-xNb alloy set in the compositions of 5 to 20 wt% of niobium. The samples were melted in an arc-melting furnace with an inert atmosphere. After this, they were characterized using optical and scanning electron microscopy, X-ray diffraction, hardness and compressive strength assays, and friction tests. The optical and scanning electron microscopy results show that the alloys have microstructure characteristics of the β phase, corroborated with X-ray diffraction, where it is impossible to observe diffraction peaks of other crystalline phases. With the increase of the niobium content, the microhardness decreases, reaching a value of 208 HV for the alloy T-15Mo-20Nb. The compressive strength of these alloys increases with the increase of the niobium content, and the Ti-15Mo-5Nb alloy has a value of 453 MPa and increases to 756 MPa in the Ti-15Mo-20Nb alloy.

Martins Jr and Grandini [15] studied a set of alloys of the Ti-15Mo-Nb system, with the alloys being prepared by arc-melting, mechanically worked by hot-rolling, homogenized, and subjected to two quenching treatments. The X-ray diffraction measurements indicated that the body-centered cubic (bcc) crystalline structure remained unchanged despite mechanical treatment or different heat treatments. The micrographs obtained by optical microscopy displayed the morphology characteristics of the β phase, with grains grown due to heat treatments. The grains were elongated and irregular after hot rolling. None of the treatments applied altered the microstructure observed after melting. These findings were consistent with the results obtained by X-ray diffraction.

In another study, Martins Jr and Grandini [16] analyzed niobium’s influence on the alloys’ structure and some mechanical properties, such as microhardness and elastic modulus, in corrosion tests and biological assays. It was observed that the crystalline structure has a bcc type, characteristic of the β phase, corroborated by the microstructure analysis. For the mechanical properties, it was observed that the increase in the niobium concentration decreases the elastic modulus, and the same occurs for microhardness. The corrosion tests showed that adding niobium did not alter the corrosion potential compared to the Ti-15Mo alloy. No cytotoxic effects were observed in all studied alloys.

Initial studies on binary titanium alloys showed that the compositions of the alloys were defined empirically [17,18]. These results showed the amount of alloying elements needed to stabilize the β phase. In the case of molybdenum, it is necessary to use at least 10 wt% for β phase stabilization, which is the best stabilizing element of this phase for biomedical alloys.

However, currently, it is possible to make the theoretical prediction about phase stabilization and the elastic modulus of titanium binary and ternary alloys (Molecular Orbital Theory) [19,20,21]. This method, called “concept d electrons”, calculates two parameters related to titanium and its alloy elements. This model involves the calculation of molecular orbitals and interactions between systems with many electrons.

Ti-50Nb is a β-titanium alloy with a high hardness value and considerably lower elastic modulus than CP-Ti and Ti-6Al-4V. Nb production is high due to Brazilian deposits that extract more than 90% of Nb’s total world production. Brazil produced 8 × 104 Ton/year (2015–2018) of Nb; in 2019, the production reached 11 × 10^4^ Ton [22]. Thus, Ti-50Nb alloy was chosen as the base for adding molybdenum.

Incorporating molybdenum into the titanium alloy (Ti-50Nb) is a deliberate and significant choice. Molybdenum, a biocompatible β-stabilizer element, introduces several advantages to a titanium alloy. Studies reveal that it bolsters corrosion resistance, diminishes the elastic modulus, and amplifies the strength [22]. These benefits accentuate the importance and relevance of our research findings to titanium alloys.

This paper presents a new Ti-50Nb-Mo system alloy (x = 0, 3, 7, and 12 wt% of molybdenum) as a biomaterial. The effect of molybdenum addition on the phase stability of Ti-50Nb-Mo system alloys was investigated as-cast and after homogenization heat treatment.

## 2. Materials and Methods

The Ti-50Nb-xMo system ingots were produced through arc-melting in an argon atmosphere to prevent gas contamination of samples. Ingots of the Ti-50Nb-xMo system (x = 0, 3, 7 and 12 wt%) were produced. The samples were separated in a nominal proportion and washed in an ultrasonic bath (SolidSteet brand) with acetone for 20 min to remove surface impurities.

To make the melting, an atmospheric cleaning process was carried out to eliminate atmospheric gases inside the fusion chamber. A mechanical vacuum pump from the EDWARDS brand was used in this process (~10^−2^ mbar). After removing the atmospheric air, inert argon gas was inserted into the fusion chamber until it reached atmospheric pressure. This cleaning process was repeated five more times to ensure an atmosphere containing only inert argon gas that would be ionized (electric arc beam) to fuse the alloy elements. More details can be seen in a previous paper by Martins Jr et al. [16].

The chemical composition analysis was performed with an EDS of Oxford, Inca 250 model. The materials were sanded on water sandpaper (#1500 mesh) and polished in alumina suspension (1 μ) for semi-quantitative EDS analyses. The EDS analyses were performed in different regions of the samples. The main parameters of the EDS analysis are a lifetime of 60.0 s and accelerating voltage = 10.00 kV.

Density measurements were carried out using an analytical balance Ohaus Explorer model and a density determination kit using Archimedes’ Principle [23]. Density measurements were carried out to verify the stoichiometry of the Ti-50Nb-Mo alloys. Such analyses were carried out on several sample pieces in different regions to verify whether the densities of the alloy produced were close to theoretical values in several sample regions.

X-ray diffraction measurements were carried out using a Rigaku, model D/Max 2100/PC, equipment with Cu-Kα radiation (λ = 1.544 Å)., current of 20 mA, the potential of 40 kV, the residence time of 1, 6 s 0.02 step size from 20° to 100° in fixed-time mode. The X-ray diffraction patterns were compared with the crystallographic records of the compact hexagonal (code: 1532765) and body-centered cubic (code: 9008554) phases. The records were obtained from the Crystallography Open Database.

For microstructural characterization, a Carl Zeiss EVO-LS15 scanning electron microscopy was used. At this stage, the metallographic preparation process for EDS analysis was repeated; however, an acidic chemical attack was carried out to visualize the topographic microstructure of the materials. This study used 200 mL of Kroll solution (15% HNO_3_, 5% HF, and 80% H_2_O) for 2 min at room temperature.

Microhardness tests were performed on a Shimadzu microdurometer. HMV model-2, with a load of 1.96N for 60 s, is based on technical standard ASTM-E384 [24]. The dynamic elasticity modulus was obtained from the impulse excitation technique based on technical standard ASTM-E1876 [25] using Sonelastic^®^ equipment from ATCP Engenharia Física. For elastic modulus measurements, the Ti-50Nb-Mo alloys were cut into sheets with dimensions of 20 × 5 × 2 mm³ (length × thickness × width).

## 3. Results

The chemical composition of the Ti-50Nb-XMo system presented in Table 1 shows the main elements found in the chemical analysis of the alloys of the Ti-50Nb-XMo system with the dispersive energy spectroscopy (EDS) technique.

It can be observed that it is in excellent agreement with the predicted amount for molybdenum, titanium, and niobium elements. This slight difference in the nominal value can be explained by minor variations in molybdenum and niobium concentration along the sample surface, and the technique used was the EDS that makes a microanalysis.

The alloys of the Ti-50Nb-XMo system are not standardized since they are alloys with unpublished chemical composition. However, several authors working with titanium alloys present similar results on the tolerance of alloy concentrations [16,26,27].

**Figure 1:**
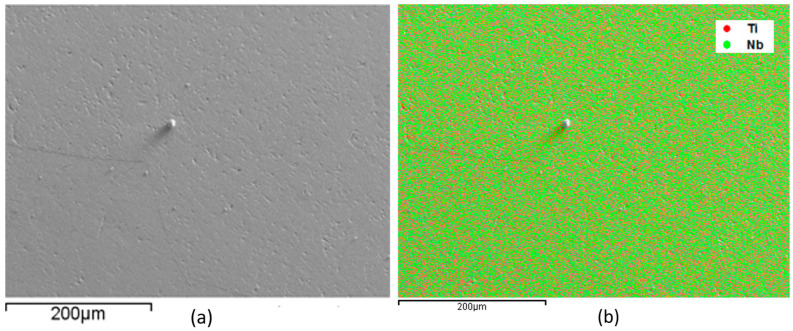
Monitoring, by EDS, of the elements that compose Ti-50Nb alloy, monitored region (**a**) and overlapping of the elements with micrograph (**b**).

**Figure 2:**
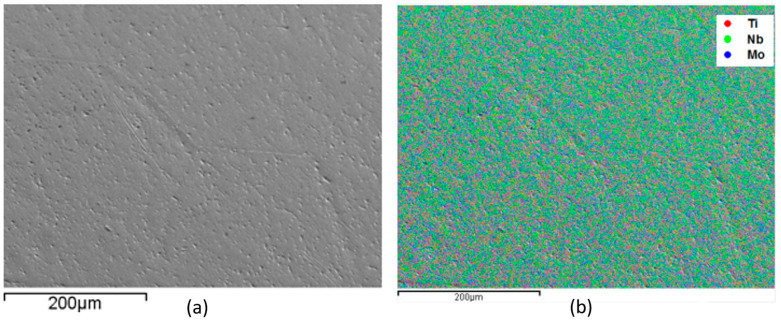
Monitoring, by EDS, of the elements that compose Ti-50Nb-3Mo alloy, monitored region (**a**) and overlapping of the elements with micrograph (**b**).

**Figure 3:**
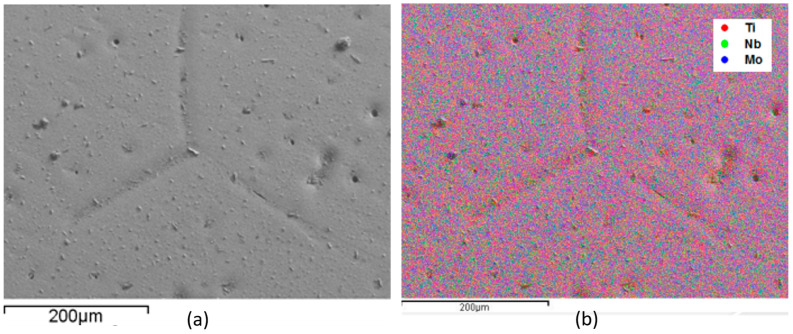
Monitoring, by EDS, of the elements that compose Ti-50Nb-7Mo alloy, monitored region (**a**) and overlapping of the elements with micrograph (**b**).

**Figure 4:**
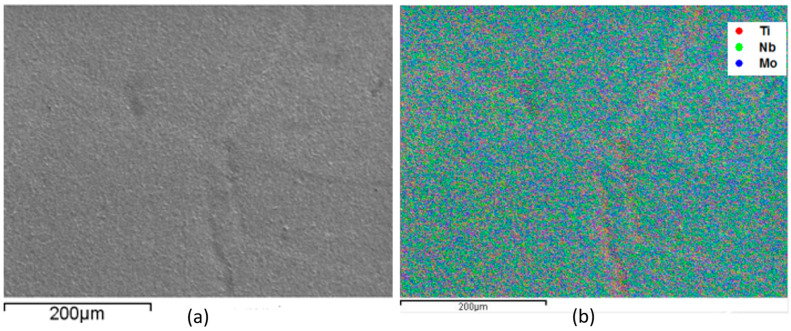
Monitoring, by EDS, of the elements that compose Ti-50Nb-12Mo alloy, monitored region (**a**) and overlapping of the elements with micrograph (**b**).

**Figure 5:**
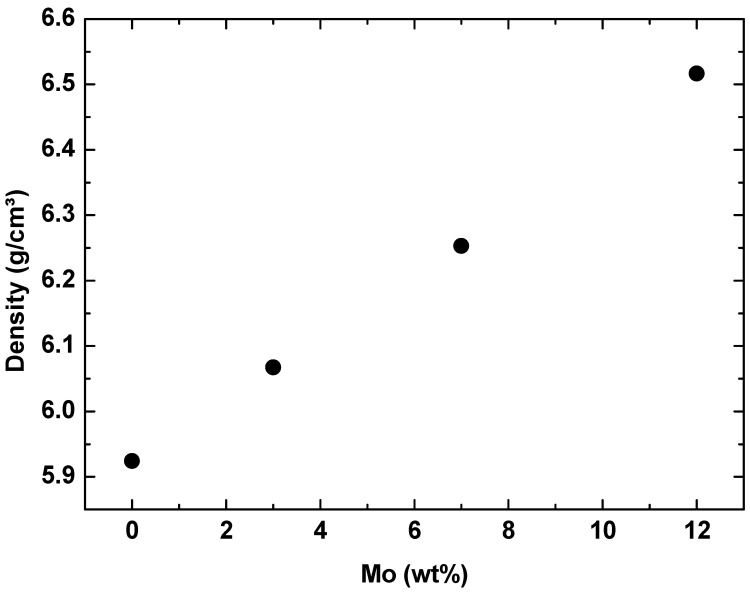
The density of the Ti–50Nb–xMo alloys as a function of molybdenum content.

EDS monitoring was performed to evaluate the sample’s alloy elements distribution. This analysis is shown in Figure 1, Figure 2, Figure 3 and Figure 4, where the clear points represent the monitored elements (red Ti, green Nb, and blue Mo). Figure 5 shows a density graph as a function of Mo concentration.

**Figure 6:**
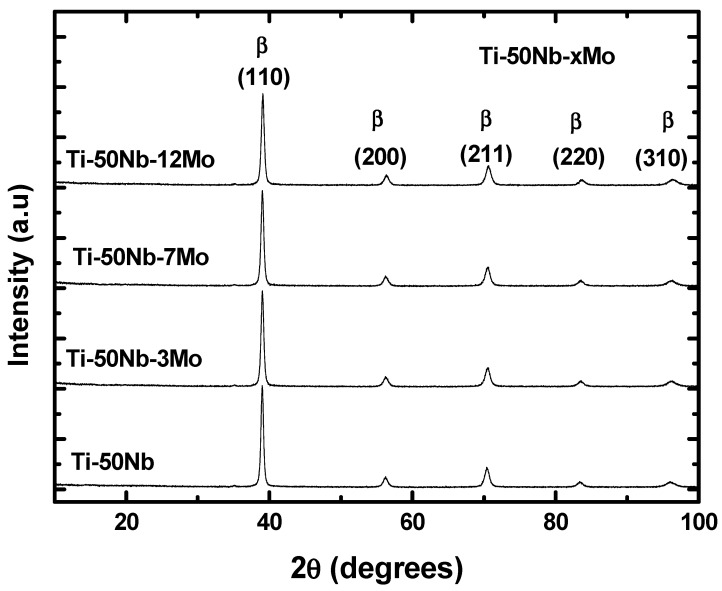
X-ray diffractograms for samples of alloys of Ti–50Nb–xMo system.

The results of X-ray diffraction measurements for Ti-50Nb-xMo system samples are shown in Figure 6. It can be observed that the diffractograms presented characteristic peaks of a cubic structure of the centered body, which is typical of the β phase of this alloy [28,29,30,31].

**Figure 7:**
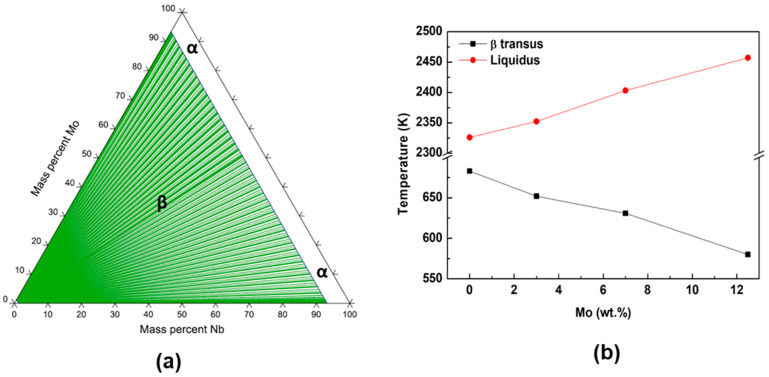
Ternary phase diagram of the Ti-Nb-Mo system alloys (**a**) and the influence of Mo content on β-transit and liquidus temperatures in Ti-50Nb-xMo system alloys (**b**).

Figure 7 is the data obtained for the alloys of the Ti-Mo-Nb system through thermodynamic simulations using the Thermo-Calc software. It presents the phase diagram of Ti-Nb-Mo alloys at atmospheric pressure and room temperature (~20 °C) (a) and the values of the β-transus and liquidus temperatures as a function of the Mo content in the Ti-50Nb alloys (b).

**Figure 8:**
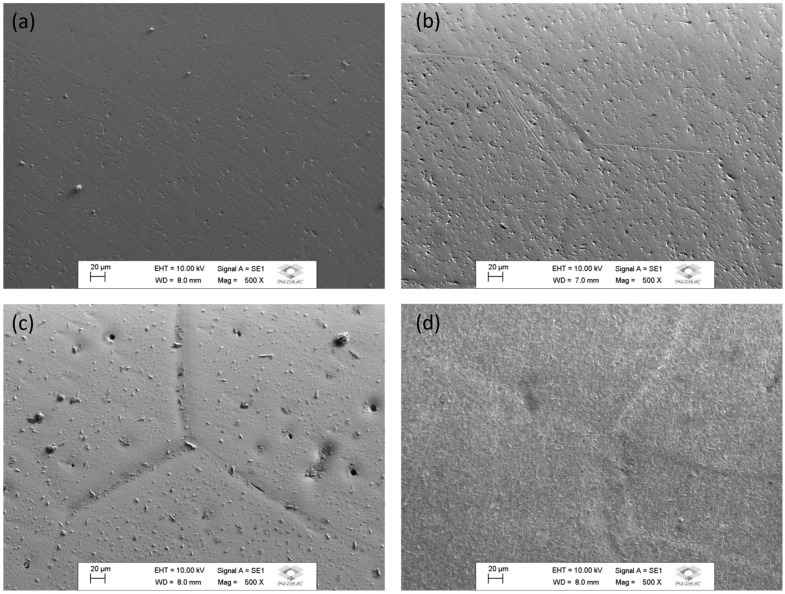
Micrographs of samples of alloys of Ti–50Nb–xMo system after melting. Ti–50Nb (**a**), Ti–50Nb–3Mo (**b**), Ti–50Nb–7Mo (**c**), Ti– 50Nb–12Mo (**d**).

Figure 8 presents the micrographs after melting; it is possible to perceive only the presence of the β phase of the Ti-50Nb-XMo system alloys, as already observed by Martins Jr et al. [16] and Cardoso et al. [32].

**Figure 9:**
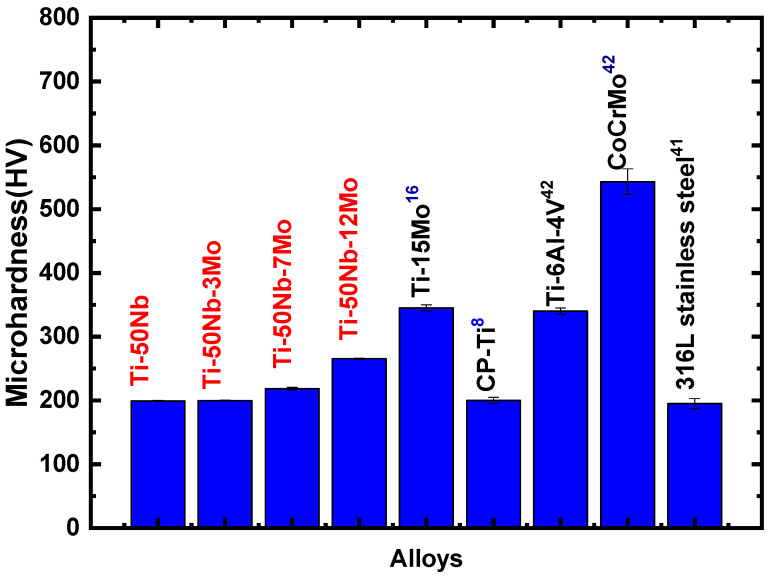
Microhardness values for Ti-50Nb-xMo system alloys (red) compared to other biomedical materials (black).

**Figure 10:**
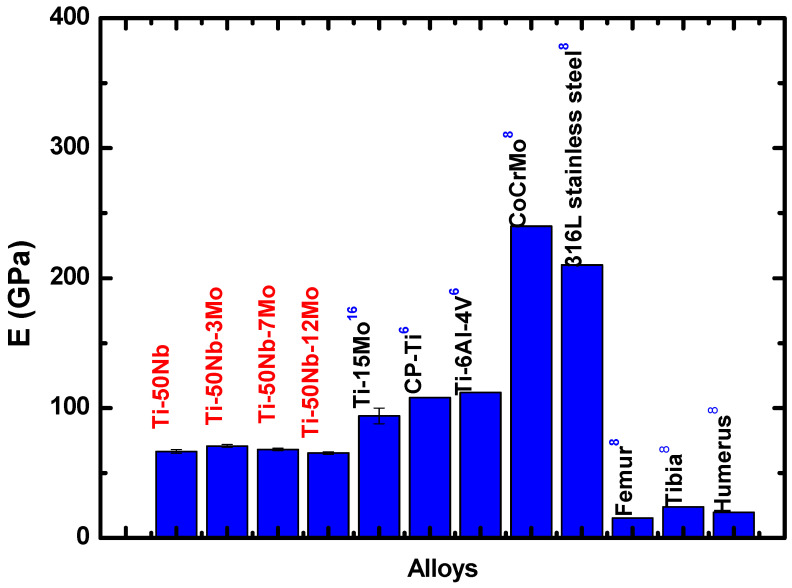
Elastic modulus values for the Ti-50Nb-xMo alloys (red) compared with alloys already used as biomaterials (black) [8].

Figure 9 shows a graph of the microhardness as a function of the molybdenum concentration. An increase in material microhardness can be observed with the increase in molybdenum concentration. This increase occurs because, with the addition of alloying elements, we decrease the movement of dislocations [17]. Figure 10 shows the elastic modulus results and compares them with alloys already used as biomaterials. The elastic modulus can be explained based on the molecular orbital theory applied to the development of β-type titanium alloys, presented by Morinaga et al. [33] and Abedel-Hady et al. [20].

**Figure 11:**
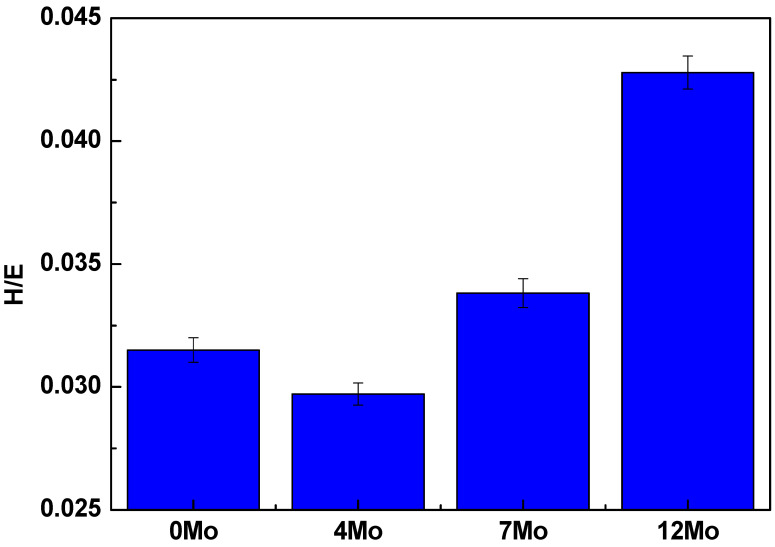
H/E ratio of the Ti–50Nb–Mo alloys as a function of Mo content.

Titanium (Ti) alloys containing β-phase tend to have the lowest elastic modulus values. Niobium and molybdenum are stabilizing elements of this phase, affecting both mechanical properties studied similarly. Therefore, the hardness to modulus (H/E) ratio is suitable for evaluating Ti alloys’ mechanical performance with Nb and Mo. The H/E ratio is directly proportional to the wear resistance of alloys, which is related to the elastic deformation of the material. Previous studies have shown that materials with an H/E ratio of at least 0.04 exhibit good wear resistance [34,35]. Figure 11 presents the H/E ratio of the Ti-50Nb-xMo alloys. The Ti–50Nb–12.5Mo alloy shows results higher than 0.04, indicating adequate wear resistance for a metallic biomaterial.

## 4. Discussion

In all figures (Figure 1, Figure 2, Figure 3 and Figure 4), part (a) shows a micrograph with 500× magnification of the analyzed sample region, and part (b) monitors the elements that make up the alloy. It is possible to observe a homogeneous distribution of alloying elements, not presenting precipitate formation, regions with higher concentrations of a given element. It is also impossible to observe precipitation, which shows that the production process of the alloys was satisfactory. This situation is very important because both niobium and molybdenum are refractory materials, and if not melted several times, sediments can be formed.

The values obtained for the density of alloys of the Ti–50Nb–Mo system are in accordance with the theoretical value [36]. Density values indicate that the alloys’ stoichiometry was achieved satisfactorily and corroborated the chemical composition results. It is possible to observe that the relationship is linear. That is, as molybdenum is added, the density is linearly increased. This increase is explained by the fact that molybdenum has a density of about 2.3 times that of titanium [36]. Even with the increase in density with adding molybdenum, the experimental density value is much lower than the density of 316L steel and CoCr, which are already used as biomaterials. This kind of material must be porous to reduce density [37].

The density of an implant can have an impact on how it interacts with the surrounding bone. Typically, implants with high density are stiffer than the human bone. This difference in stiffness can create a mismatch and lead to increased stress points around the implant, which can pose a significant risk to the success of the implantation. Moreover, introducing a very dense implant can interfere with the natural adaptation of the bone to the applied forces, which can result in problems such as bone loss around the implant.

The X-ray diffractograms can obtain a series of information. The position of the peaks provides us with information on the dimensions of the unit cell, the crystalline system, and the identification of the crystalline phases. Based on this, it was identified that all alloys have β structure as observed [38,39].

From the X-ray diffraction patterns, it is possible to calculate the lattice parameters of the BCC crystal structure by Bragg’s law, as shown by [40]. The results indicated that the Ti-50Nb alloy has a lattice parameter of approximately 3.278 ± 0.008 Å. When adding the element Mo (0.1363 nm), which has a smaller atomic radius than Nb (0.1430 nm), a contraction of the BCC structure was expected, and this behavior was achieved in the Ti-50Nb-Mo alloys.

The addition of Mo slightly reduced the lattice parameters of the Ti-50Nb-Mo alloys, where the alloy with 3Mo has a value of 3.275 ± 0.005 Å; 7Mo with 3.274 ± 0.004 Å; and finally, the alloy with 12Mo has the lowest lattice parameter of all the alloys produced in this study, with a value of approximately 3.271 ± 0.007 Å.

It is observed that there are no significant changes in the values of the lattice parameters with the addition of Mo in the Ti-50Nb alloys, as the alloys are of the stable β type.

The Thermo-Calc simulations showed that the Ti-50Nb-Mo alloys are of the β type, where the ternary diagram produced showed a broad β field region (green) for high concentrations of Nb and Mo. In addition, β-transus temperature and liquid temperature were also calculated by the software. A decrease in the β-transus temperature and an increase in the liquid temperature can be observed by adding Mo to the alloys. The behavior of the drop in β-transus temperature was expected because the β-stabilizing character of Mo is widely understood in the literature. Regarding liquid temperature, in the Ti-50Nb-Mo system, Mo acts as a substitutional element for Ti, where Mo has a higher melting point than Ti, increasing the liquid temperature of alloys with higher concentrations of Mo.

In all scanning electron microscopy images, the characteristics of the β phase can be observed as the contours of well-defined grains (In Ti alloys, defined grain boundaries are characteristic of β-type alloys). Thus, the micrographs are following the results of X-ray diffraction and Molecular Orbital Theory [21,33]; it is observed that the alloys used are in the β field.

Currently, in producing new titanium alloys, the scientific community seeks to produce β-type alloys, as these materials tend to have a lower elastic modulus value than α-type alloys [28], which would increase the useful life of a biomedical implant, avoiding the failure effect known as “stress shielding.” Furthermore, α-type alloys have a higher atomic packing factor (compact hexagonal structure) than β-type alloys, which can result in a high hardness value due to the reduction in atomic interstices, making handling difficult for the forging parts, such as screws and plates orthopedic.

Comparing the data with the literature, Figure 9, it can be seen that the alloys of the Ti-50Nb-xMo system have a microhardness less than or equal to that of CP-Ti [38] and 316L stainless steel [41], both materials that already have biomedical applications. On the other hand, when molybdenum is added, the values increase. Ti-6Al-4V and Co-Cr-Mo alloy [42] have much higher microhardness values than those studied, showing that the materials developed in this work are promising materials in the biomedical area. This decrease in microhardness is very interesting, as it facilitates the mechanical conformation of these alloys.

Regarding the elastic modulus values, it is observed that there are no significant changes with the addition of Mo, with the alloys produced having approximately 68 GPa. As observed in the results of structural characterization using the X-ray diffraction technique, there are no changes in crystalline structures; that is, the alloys are of the stable β type. Furthermore, there are also no significant changes in the values of the lattice parameters, which would influence the elastic modulus results, as β type alloys with dilated lattice parameters (due to temperature, mechanical deformation, and addition of solutes and interstitials) tend to have lower elastic modulus values due to the decrease in interatomic forces.

The decrease in the elastic modulus of the Ti-50Nb-XMo system follows the Molecular Orbital Theory. The elastic modulus values found show that these alloys are promising materials for biomedical applications since they have low elastic modulus values when compared with Ti-6Al-4V alloy and titanium cp. In addition, the values are about 60% lower than stainless steel and 70% lower than Co-Cr-Mo alloys [6].

Thus, it can be observed that the elastic modulus of the alloys studied in this work is closer to the human bone than the traditional alloys used [6,30,43,44,45,46]. It is essential to highlight that a low modulus value is required to produce new metals for biomedical use to avoid the “stress shielding” effect. By implanting a material with high values of modulus of elasticity, the implant shields the bone from mechanical loads that are fundamental to keeping the structure healthy, causing a decrease in bone density [47,48,49].

## 5. Conclusions

Based on the results obtained in this work, it can be concluded:

- The chemical composition results showed that the alloys produced in this study have good quality. In contrast, the experimental values obtained by the semi-quantitative EDS technique showed that the alloy elements (Ti, Nb, and Mo) have values close to the nominal ones. Furthermore, the density results showed that the alloys have suitable stoichiometry.
- The densities of Ti-50Nb-Mo alloys increase with the Mo content, with Mo having a higher density value than Ti.
- All alloys have the body-centered cubic crystal structure, β, confirmed by X-ray diffraction, microscopy techniques, and thermo-calc simulation results.
- The results show that the addition of molybdenum decreased elastic modulus.
- The values found show such alloys as excellent candidates for biomaterial use.
- However, among the alloys studied, the one with the best properties was the Ti-50Nb-12Mo alloy because it presented a lower elastic modulus and H/E ratio.

## Figures and Tables

**Table 1:** Chemical composition of the Ti–50Nb–xMo alloys after melting.

Element (Wt%)	Ti-50Nb	Ti-50Nb-3Mo	Ti-50Nb-7Mo	Ti-50Nb-12Mo
Ti	balance	balance	balance	balance
Nb	54.5 ± 0.2	55.0 ± 0.3	54.9 ± 0.2	51.2 ± 0.2
Mo	<0.01	2.9 ± 0.3	7.6 ± 0.3	12.7 ± 0.3

## Data Availability

The data presented in this study are available on request from the corresponding author.
